# Steatosis paradox: Unraveling pathways of suppressive effect of hepatic steatosis on hepatitis B virus

**DOI:** 10.1016/j.bj.2026.100969

**Published:** 2026-03-25

**Authors:** Shang-Chin Huang, Jia-Horng Kao

**Affiliations:** a Department of Internal Medicine, National Taiwan University Hospital Bei-Hu Branch, Taipei, Taiwan; b Division of Gastroenterology and Hepatology, Department of Internal Medicine, National Taiwan University Hospital, Taipei, Taiwan; c Hepatitis Research Center, National Taiwan University Hospital, Taipei, Taiwan; d Graduate Institute of Clinical Medicine, National Taiwan University College of Medicine, Taipei, Taiwan

**Keywords:** Hepatic steatosis, Hepatitis B virus, Fatty liver, Adiponectin, Functional cure

## Abstract

The global intersection of chronic hepatitis B (CHB) and metabolic dysfunction-associated steatotic liver disease (MASLD) has revealed an intriguing clinical paradox: hepatic steatosis is frequently associated with lower hepatitis B virus (HBV) viral loads, higher rates of HBsAg seroclearance, and even favorable clinical outcomes. Accumulating data suggest that lipid accumulation transforms hepatocytes into a hostile microenvironment for HBV through at least four pathways, including immune modulation, metabolic reprogramming, endoplasmic reticulum stress, and impaired autophagy. Deciphering these molecular pathways not only explains the unique natural history of CHB patients with concurrent MASLD but also highlights novel therapeutic targets such as selective modulators of cellular stress that could complement existing antivirals or immune enhancers to facilitate functional cure. In this review, the current evidence regarding the cellular mechanisms underlying the suppressive effect of steatosis on HBV is summarized, providing a translational roadmap for exploiting these virus-host metabolic interactions to develop next-generation, novel host-targeting therapies against HBV.

## 1 Introduction

Chronic hepatitis B (CHB) has been an important global infectious disease affecting more than 300 million individuals worldwide, leading to cirrhosis, hepatocellular carcinoma (HCC), and liver-related mortality [1]. Despite the widespread availability of vaccination and antiviral therapies [2], many patients continue to experience long-term complications driven by persistent viral activity and host-virus interactions. At the same time, the global surge of metabolic dysfunction-associated steatotic liver disease (MASLD, previously termed non-alcoholic fatty liver disease, NAFLD) has introduced a new dimension of complexity into chronic liver disease [3,4]. It is now estimated that around 30% of CHB patients have concomitant hepatic steatosis, reflecting overlapping epidemics of viral hepatitis and metabolic disorders in both Asian and the Western populations [5].

Clinical observations over the past decade have revealed a paradoxical interaction between hepatic steatosis and hepatitis B virus (HBV) infection. Several cohort studies consistently reported that CHB patients with concomitant steatosis exhibit lower serum HBV DNA and lower proportion of hepatitis B e antigen (HBeAg) positivity [6], higher rates of hepatitis B surface antigen (HBsAg) seroclearance [[7], [8], [9], [10]], and in particular cohorts, reduced risks of HCC, cirrhosis, and liver-related mortality compared to those without steatosis [6,8,9,[11], [12], [13], [14], [15]]. These findings stand in contrast to the general notion that steatosis may accelerate disease progression in other chronic liver conditions.

It is crucial to elucidate how and why hepatic steatosis modifies the natural course of CHB. This review aims to summarize current evidence on this paradoxical clinical relationship and to explore the potential molecular and cellular mechanisms that may explain how steatosis influences HBV replication, disease progression, and HCC risks.

## 2 Impact of hepatic steatosis on the natural history of CHB

The interaction between hepatic steatosis and CHB outcomes has been a subject of ongoing debate. Historically, some studies indicated that concurrent fatty liver disease might exacerbate liver injury, thereby accelerating fibrosis progression and increasing the risk of HCC [[16], [17], [18]], particularly when the confounding effects of systemic metabolic dysfunctions were not fully adjusted. However, emerging data have challenged this view; several lines of evidence have consistently shown that hepatic steatosis exerts a distinct influence on the natural history of CHB. Multiple longitudinal cohorts demonstrated that CHB patients with concurrent steatosis experienced a higher likelihood of HBsAg seroclearance, compared to those without steatosis [[7], [8], [9], [10]]. Moreover, these patients tended to show lower rates of cirrhosis, HCC, and mortality, even after adjustment for viral and metabolic confounders [6,8,9,[11], [12], [13], [14], [15],^19^]. For instance, large-scale real-world data reported approximately 55% lower risk of HCC among CHB patients with steatosis [6], suggesting that steatosis may modulate the viral-host equilibrium toward a less aggressive disease course.

Importantly, this phenomenon appears to be unique to hepatic steatosis itself and is not reproduced by other components of metabolic dysfunction. Clinical analyses found that metabolic dysfunctions, such as diabetes mellitus (DM), exerted the opposite effect, increasing HCC risks and accelerating fibrosis progression in CHB [[19], [20], [21], [22], [23], [24], [25], [26], [27]]. In a large cohort study conducted in 1076 CHB patients undergoing liver biopsy, metabolic dysfunction-associated fatty liver disease (MAFLD) was associated with an increased risk of liver-related clinical events, but the presence of steatohepatitis was not a significant factor, highlighting the detrimental effect of metabolic dysfunctions instead of steatosis [17]. In 330 prospectively collected CHB patients with low viremia, fatty liver disease (without adjustment for metabolic dysfunctions) was associated with a higher chance of function cure as well as worsening liver fibrosis [7]. These contrasting trends imply that steatosis per se, rather than systemic metabolic impairment, plays a distinct biological role in HBV replication [8,9]; the two diagnostic components of MASLD (hepatic steatosis and cardiometabolic risk factors) have divergent impacts on the natural history of CHB.

**Fig. 1:**
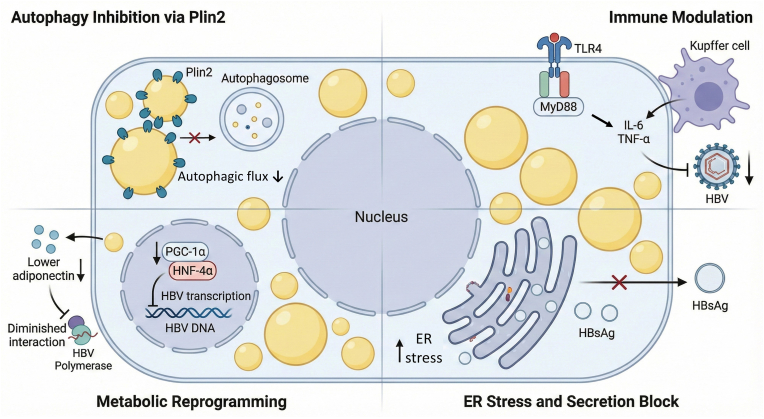
**Possible mechanisms underlying the inhibitory effect of hepatic steatosis on the HBV life cycle.** This schematic illustrates a steatotic hepatocyte where lipid accumulation exerts a multifaceted suppressive effect on HBV replication and secretion. (A) Immune Modulation: SFA activate TLR4 signaling on the cell membrane, triggering the release of antiviral cytokines (IL-6 and TNF-α) that inhibit HBV replication. (B) Metabolic Reprogramming: In the nucleus, the expression or activity of PGC-1α is downregulated in the steatotic state, leading to reduced co-activation of HNF-4α and diminished HBV transcriptional drive. Additionally, reduced adiponectin levels further weaken the interaction between the HBV Polymerase and pgRNA. (C) ER Stress and Secretion Block: Lipid overload induces ER stress. This stress response impairs the ER's capacity to fold and traffic viral proteins, causing HBsAg and virions to be retained intracellularly. (D) Autophagy Inhibition: Upregulated Plin2 on the surface of lipid droplets stabilizes lipids and inhibits autophagic flux. This blockade prevents HBV from utilizing the autophagic machinery for nucleocapsid maturation and egress. Abbreviations: HBV, hepatitis B virus; SFA, saturated fatty acids; TLR4, Toll-like receptor 4; TNF-α, tumor necrosis factor-α; PGC-1α, peroxisome proliferator-activated receptor-γ coactivator-1α; HNF-4α, hepatocyte nuclear factor-4α; ER, endoplasmic reticulum; Plin2, Perilipin 2.

Such an unexpected dissociation between metabolic and viral outcomes raises important and interesting mechanistic questions. Exploring why lipid accumulation in hepatocytes, typically regarded as a pathological hallmark, correlates with reduced HBV activity and improved liver-related outcomes may help identify novel biological pathways and potential therapeutic targets against HBV [28]. The following sections will summarize current lines of evidence on these mechanistic links (see Fig. 1).

## 3 Possible pathways

### 3.1 Immune Modulation

Hepatic steatosis substantially reshapes the hepatic immune microenvironment, and many data suggest that these immune alterations may contribute to the suppression of HBV replication. Saturated fatty acids can activate Toll-like receptor 4 (TLR4) signaling on Kupffer cells and hepatocytes, leading to MyD88-dependent and -independent induction of antiviral cytokines such as IL-6 and TNF-α [29,30] In HBV transgenic mice and steatotic HepG2.2.15 cells, this TLR4 activation is associated with reduced HBsAg, HBeAg, and HBV DNA levels. In these models, upregulation of TLR4 and MyD88 is accompanied by increased IL-6 and TNF-α expression, which in turn may reduce HBV expression [31].

In addition to cytokine-mediated antiviral signaling, steatohepatitis is closely linked to Fas-mediated apoptosis. In human NASH, hepatocyte apoptosis and Fas expression are markedly increased compared with controls, indicating that lipid-laden hepatocytes are more susceptible to death receptor-mediated apoptosis [32]. In another study, steatohepatitis unconventionally activated CD8^+^PD1^+^ T cells in the liver, leading to hepatocyte damage [33]. Although these data were derived from NASH cohorts, they provided a biologic basis for enhanced clearance of injured or infected hepatocytes in steatotic livers, which could secondarily reduce the intrahepatic HBV reservoir.

Taken together, these findings support a model in which steatosis-driven immune modulation, creating a hepatic milieu that is unfavorable for HBV persistence.

### 3.2 Metabolic reprogramming and adipokine dysregulation

Beyond immune modulation, hepatic steatosis profoundly reprograms hepatocellular metabolism in ways that may be unfavorable for HBV replication. In patients with concurrent CHB and MASLD, lipid accumulation reflects an over-nutrition state characterized by altered glucose and lipid fluxes, and changes in adipokine profiles. Several clinical and translational studies suggest that such steatosis-driven metabolic reprogramming contributes to the observed inverse association between steatosis and HBV activity [[34], [35], [36], [37], [38]].

A central node in this axis is peroxisome proliferator-activated receptor-γ coactivator-1α (PGC-1α), a nutrient-sensitive coactivator that integrates fasting signals to drive gluconeogenesis and fatty acid oxidation, in part via coactivation of HNF-4α [34]. Experimental studies demonstrated that PGC-1α served as an adaptor molecule interacting with enhancer I and II region of HBV genome, thereby facilitating HBV production [34,39,40]. Notably, hepatic PGC-1α expression is frequently negatively correlated with MASLD severity, reflecting impaired oxidative metabolism in steatotic hepatocytes [41]. It is thus plausible that in CHB patients with steatosis, reduced PGC-1α activity may lead to diminished HBV transcriptional drive, contributing to lower HBV DNA and HBsAg levels.

Steatosis is also accompanied by adipokine dysregulation, which may further influence HBV replication. Among these, adiponectin is particularly relevant. A study showed that adiponectin, a downstream target of PPAR-γ, could enhance HBV replication, potentially by strengthening interactions between the HBV P protein and pgRNA or DNA complexes [42]. In the presence of hepatic steatosis, serum adiponectin levels are often reduced [43]; on the other hand, adiponectin could potentially diminish steatohepatitis [44]. Taken together, concurrent hepatic steatosis may be associated with a lower level of adiponectin, leading to HBV suppression and a better prognosis [38].

Collectively, these findings support a model in which hepatic steatosis reshapes the metabolic landscape, resulting in a hepatocellular state that is metabolically less permissive for HBV replication.

### 3.3 Endoplasmic reticulum stress

Hepatic steatosis is a well-recognized trigger of endoplasmic reticulum (ER) stress, and a growing body of evidence indicates that ER stress can alter the HBV life cycle. In steatotic hepatocytes, excess saturated fatty acids and lipid droplet expansion disturb ER homeostasis [45]. This stress-adaptive program modifies protein synthesis, trafficking, and degradation in ways that can interfere with HBV protein production and virion secretion [46].

A key piece of direct experimental evidence comes from a study in HBV-producing HepG2.2.15 cells, where oleic acid and stearic acid were used to induce ER stress. This study showed that ER stress, confirmed by upregulation of GRP78, p-PERK and *p*-eIF2α, inhibited the secretion of HBsAg and HBV DNA into the supernatant, while simultaneously causing accumulation of HBsAg and HBV DNA inside hepatocytes. Pharmacologic alleviation of ER stress with 4-phenylbutyric acid partially restored viral secretion [47]. In another cellular study, under entecavir treatment, ER stress accelerated extracellular HBV DNA clearance but delayed intracellular HBV DNA clearance, highlighting that ER stress primarily acts by blocking viral secretion rather than uniformly suppressing replication [48].

These studies indicated that in the context of steatosis-related ER stress, HBV may encounter a hostile secretory environment. Clinically, this provides a plausible mechanistic link between steatosis, lower circulating HBV DNA/HBsAg levels, and altered natural history. More mechanistic studies are warranted to confirm the detailed pathways.

### 3.4 Autophagy and the lipid-droplet/perilipin-2 axis

Autophagy, the process by which cells degrade and recycle cytoplasmic contents including damaged organelles and lipid droplets, plays an important role in the interplay between hepatic steatosis and HBV replication. Autophagic flux are essential for enhancing HBV viral persistence [49,50]. Lipid droplet accumulation is a hallmark of hepatic steatosis, and the surface protein perilipin 2 (Plin2) has emerged as a key regulator of lipid droplet stability and autophagic flux [51].

An in vitro study showed that in HepG2.2.15 cells stimulated with palmitic and oleic acids, Plin2 expression increased significantly, autophagy-related protein levels decreased, and HBV DNA, HBsAg and HBeAg levels were suppressed. Importantly, knock-down of Plin2 reversed the suppression of HBV markers, and addition of the autophagy inhibitor 3-methyladenine re-suppressed HBV DNA replication, thus demonstrating that steatosis inhibits HBV replication via the Plin2-autophagy pathway [52].

Mechanistically, Plin2-mediated stabilization of lipid droplets prevents their catabolism via lipophagy, thereby reducing free fatty acid flux into lysosomes [51]. Thus, in steatotic hepatocytes, decreased autophagic flux impairs HBV's ability to exploit autophagosomes for nucleocapsid maturation and virion egress. This mechanistic insight aligns with broader reviews indicating that autophagy supports HBV replication [49,50], and conversely, its inhibition can reduce HBV particle formation.

From a clinical perspective, this autophagy-lipid droplet axis offers a plausible link between hepatic steatosis and lower HBV viral burden. These mechanistic findings complement the immune modulation and metabolic reprogramming pathways previously discussed, and set the stage for exploring how these cellular stress responses integrate to modulate HBV persistence and hepatocarcinogenesis risk.

## 4 Perspectives and future directions

### 4.1 Translating mechanistic insights into novel therapeutics

The elucidation of the cellular interplay between hepatic steatosis and HBV life cycle offers more than just academic curiosity; it reveals a potential treasure trove of druggable targets for functional cure strategies. As summarized in this review, the steatotic hepatocytes create a hostile environment for HBV through distinct pathways. The important question for future drug development is whether we can mimic these virus-suppressive effects without inducing the pathological lipotoxicity associated with actual fatty liver disease. For instance, therapeutic agents that selectively modulate PGC-1α activity or intervene in the Plin2-autophagy pathway could potentially suppress HBV transcriptional drive or nucleocapsid maturation [34,52]. Similarly, induction of the ER stress has been shown to interfere with HBsAg secretion [47]. If we could uncouple the antiviral properties of these stress responses from their deleterious effects on hepatocyte viability, we may identify a new class of host-targeting antivirals that complement existing nucleos(t)ide analogues.

### 4.2 The "double-edged sword" dilemma

While the mechanisms detailed above provide a biological basis for the favorable viral outcomes such as HBsAg seroclearance in steatotic patients, clinicians must navigate a "double-edged sword" dilemma [53]. It is imperative to distinguish between the local antiviral effects of steatosis per se and the systemic harm caused by metabolic dysfunctions. Although steatosis may suppress viral replication, the broader spectrum of metabolic dysfunctions synergistically drives liver fibrosis progression and increases the risks of HCC as well as mortality [54,55]. Therefore, the clinical management of CHB patients with concurrent MASLD should focus on correcting these metabolic risk factors. The proven long-term benefits of controlling cardiovascular risk and preventing metabolic-driven fibrosis may outweigh the effect of viral suppression by steatosis.

Additionally, the paradigm for assessing CHB patients must evolve to a holistic care. Prognostic models should no longer rely solely on viral markers such as HBV DNA but must integrate a comprehensive metabolic profile. Active management of metabolic risk factors is essential, not only to reduce non-liver morbidity but to mitigate the synergistic oncogenic effects of metabolic and viral insults.

### 4.3 Unraveling the mysteries left

Despite the progress made in mapping these pathways, our understanding remains incomplete. The current mechanistic models, ranging from lipid-mediated immune activation to organelle stress responses, likely operate in concert rather than in isolation. However, it remains unclear which of these pathways is dominant in vivo, or how they vary across different HBV genotypes and stages of liver fibrosis. Furthermore, it is yet to be determined whether the hostile environment created by steatosis leads to the permanent silencing of cccDNA or merely a transient suppression of viral protein production.

Future research directions should bridge the gap between these molecular observations and clinical phenotypes. Only by fully resolving these molecular enigmas can we hope to harness the "steatosis paradox" for therapeutic gain, turning a metabolic burden into a strategic weapon against HBV persistence.

## 5 Conclusions

The interactions between hepatic steatosis and CHB represent a fascinating clinical paradox. Unlike other metabolic comorbidities that typically accelerate liver disease progression, steatosis per se exerts a unique suppressive effect on HBV replication. As summarized in this review, this phenomenon is driven by multifaceted cellular responses that transform the hepatocytes into a hostile microenvironment for the virus. However, our current understanding of these mechanisms remains fragmentary, and further research is required to fully unravel how these pathways integrate in vivo. Deciphering these molecular complexities offers a strategic opportunity: if we can uncouple the antiviral mechanisms of cellular stress from their lipotoxic consequences, we may pave the way for a new class of host-targeting therapeutics. Such agents could selectively exploit these suppressive pathways to complement existing treatments, ultimately bringing us closer to the ultimate goal of HBV cure.

## Authors’ contribution

SC Huang: drafting of the manuscript.

JH Kao: critical revision for important intellectual content.

## Declaration of AI-assisted technologies in the writing process

During the preparation of this work, the authors used *Grammarly* and *ChatGPT* for English proofreading and editing, and *Gemini* for assisting in drafting the Figure. After using these tools, the authors reviewed and edited the content as needed and take full responsibility for the content of the publication.

## Funding

This paper was not funded.

## Declaration of competing interest

S.-C. H. was on speaker's bureau for Gilead Sciences.

J.-H. K. has served as a consultant for Abbvie, Abbott, Gilead Sciences, Roche, and Sysmex and on speaker's bureaus for Abbvie, Bristol-Myers Squibb, Gilead Sciences, Merck Sharp and Dohme, and Sysmex.

## References

[1] (2022). Global, regional, and national burden of hepatitis B, 1990-2019: a systematic analysis for the global burden of disease study 2019. Lancet Gastroenterol Hepatol.

[2] Huang SC, Kao JH. (2025). Combining therapeutic agents to target the immune systems of hepatitis B patients: what do we need to consider?. Expet Rev Gastroenterol Hepatol.

[3] Tilg H, Petta S, Stefan N (2026). Metabolic dysfunction-associated steatotic liver disease in adults: a review. JAMA.

[4] Younossi ZM, Zelber-Sagi S, Lazarus JV (2025). Global consensus recommendations for metabolic dysfunction-associated steatotic liver disease and steatohepatitis. Gastroenterology.

[5] Jiang D, Chen C, Liu X (2021). Concurrence and impact of hepatic steatosis on chronic hepatitis B patients: a systematic review and meta-analysis. Ann Transl Med.

[6] Huang SC, Su TH, Tseng TC (2023). Distinct effects of hepatic steatosis and metabolic dysfunction on the risk of hepatocellular carcinoma in chronic hepatitis B. Hepatol Int.

[7] Mak LY, Hui RW, Fung J (2020). Diverse effects of hepatic steatosis on fibrosis progression and functional cure in virologically quiescent chronic hepatitis B. J Hepatol.

[8] Li J, Yang HI, Yeh ML (2021). Association between fatty liver and cirrhosis, hepatocellular carcinoma, and hepatitis B surface antigen seroclearance in chronic hepatitis B. J Infect Dis.

[9] Wong YJ, Nguyen VH, Yang HI (2023). Impact of fatty liver on long-term outcomes in chronic hepatitis B: a systematic review and matched analysis of individual patient data meta-analysis. Clin Mol Hepatol.

[10] Huang SC, Su TH, Tseng TC (2024). Metabolic dysfunction-associated steatotic liver disease facilitates hepatitis B surface antigen seroclearance and seroconversion. Clin Gastroenterol Hepatol.

[11] Huang SC, Su TH, Tseng TC (2025). Pre-existing and new-onset metabolic dysfunctions increase cirrhosis and its complication risks in chronic hepatitis B. Am J Gastroenterol.

[12] Huang SC, Su TH, Tseng TC (2025). All-cause and cause-specific mortality in patients with chronic hepatitis B and concurrent steatotic liver disease. J Hepatol.

[13] Hsueh RC, Wu WJ, Lin CL (2022). Impact of PNPLA3 p.I148M and hepatic steatosis on long-term outcomes for hepatocellular carcinoma and HBsAg seroclearance in chronic hepatitis B. J Hepatocell Carcinoma.

[14] Mak LY, Hui RW, Fung J (2021). Reduced hepatic steatosis is associated with higher risk of hepatocellular carcinoma in chronic hepatitis B infection. Hepatol Int.

[15] Oh JH, Lee HW, Sinn DH (2021). Controlled attenuation parameter value and the risk of hepatocellular carcinoma in chronic hepatitis B patients under antiviral therapy. Hepatol Int.

[16] Lee YB, Ha Y, Chon YE (2019). Association between hepatic steatosis and the development of hepatocellular carcinoma in patients with chronic hepatitis B. Clin Mol Hepatol.

[17] van Kleef LA, Choi HSJ, Brouwer WP (2021). Metabolic dysfunction-associated fatty liver disease increases risk of adverse outcomes in patients with chronic hepatitis B. JHEP Rep.

[18] Mao X, Cheung KS, Peng C (2023). Steatosis, HBV-related HCC, cirrhosis, and HBsAg seroclearance: a systematic review and meta-analysis. Hepatology.

[19] Huang R, Jun DW, Toyoda H (2025). Impacts of metabolic syndrome diseases on long-term outcomes of chronic hepatitis B patients treated with nucleos(t)ide analogues. Clin Mol Hepatol.

[20] Huang YW, Wang TC, Lin SC (2013). Increased risk of cirrhosis and its decompensation in chronic hepatitis B patients with newly diagnosed diabetes: a nationwide cohort study. Clin Infect Dis.

[21] Fu SC, Huang YW, Wang TC (2015). Increased risk of hepatocellular carcinoma in chronic hepatitis B patients with new onset diabetes: a nationwide cohort study. Aliment Pharmacol Ther.

[22] Hsiang JC, Gane EJ, Bai WW (2015). Type 2 diabetes: a risk factor for liver mortality and complications in hepatitis B cirrhosis patients. J Gastroenterol Hepatol.

[23] Yang C, Wan M, Lu Y (2022). Associations between diabetes mellitus and the risk of hepatocellular carcinoma in Asian individuals with hepatitis B and C infection: systematic review and a meta-analysis of cohort studies. Eur J Cancer Prev.

[24] Campbell C, Wang T, McNaughton AL (2021). Risk factors for the development of hepatocellular carcinoma (HCC) in chronic hepatitis B virus (HBV) infection: a systematic review and meta-analysis. J Viral Hepat.

[25] Wong GL, Wong VW, Choi PC (2009). Metabolic syndrome increases the risk of liver cirrhosis in chronic hepatitis B. Gut.

[26] Wong GL, Chan HL, Yu Z (2014). Coincidental metabolic syndrome increases the risk of liver fibrosis progression in patients with chronic hepatitis B--a prospective cohort study with paired transient elastography examinations. Aliment Pharmacol Ther.

[27] Li J, Xu L, Rui F (2025;83(5):1273-86.). Type 2 diabetes mellitus as an independent predictor of significant fibrosis in treatment-naïve chronic hepatitis B patients with concurrent hepatic steatosis. Hepatology.

[28] Huang SC, Liu CJ. (2023). Chronic hepatitis B with concurrent metabolic dysfunction-associated fatty liver disease: challenges and perspectives. Clin Mol Hepatol.

[29] Spruss A, Kanuri G, Wagnerberger S (2009). Toll-like receptor 4 is involved in the development of fructose-induced hepatic steatosis in mice. Hepatology.

[30] Tourkochristou E, Assimakopoulos SF, Thomopoulos K (2022). NAFLD and HBV interplay - related mechanisms underlying liver disease progression. Front Immunol.

[31] Zhang RN, Pan Q, Zhang Z (2015). Saturated fatty acid inhibits viral replication in chronic hepatitis B virus infection with nonalcoholic fatty liver disease by toll-like receptor 4-Mediated innate immune response. Hepat Mon.

[32] Feldstein AE, Canbay A, Angulo P (2003). Hepatocyte apoptosis and fas expression are prominent features of human nonalcoholic steatohepatitis. Gastroenterology.

[33] Pfister D, Núñez NG, Pinyol R (2021). NASH limits anti-tumour surveillance in immunotherapy-treated HCC. Nature.

[34] Shlomai A, Paran N, Shaul Y. (2006). PGC-1alpha controls hepatitis B virus through nutritional signals. Proc Natl Acad Sci U S A.

[35] Chu CM, Lin DY, Liaw YF. (2007). Does increased body mass index with hepatic steatosis contribute to seroclearance of hepatitis B virus (HBV) surface antigen in chronic HBV infection?. Int J Obes.

[36] Zhang Z, Pan Q, Duan XY (2012). Fatty liver reduces hepatitis B virus replication in a genotype B hepatitis B virus transgenic mice model. J Gastroenterol Hepatol.

[37] Hui RWH, Seto WK, Cheung KS (2018). Inverse relationship between hepatic steatosis and hepatitis B viremia: results of a large case-control study. J Viral Hepat.

[38] Chen CL, Yang WS, Yang HI (2025). Plasma Adiponectin levels in relation to chronic hepatitis B infection progression to liver cancer milestones: a prospective study. Liver Cancer.

[39] Shalaby RE, Iram S, Çakal B (2017). PGC1α transcriptional adaptor function governs hepatitis B virus replication by controlling HBcAg/p21 protein-mediated capsid formation. J Virol.

[40] Li X, Pan E, Zhu J (2018). Cisplatin enhances hepatitis B virus replication and PGC-1α expression through endoplasmic reticulum stress. Sci Rep.

[41] Piccinin E, Villani G, Moschetta A. (2019). Metabolic aspects in NAFLD, NASH and hepatocellular carcinoma: the role of PGC1 coactivators. Nat Rev Gastroenterol Hepatol.

[42] Yoon S, Jung J, Kim T (2011). Adiponectin, a downstream target gene of peroxisome proliferator-activated receptor γ, controls hepatitis B virus replication. Virology.

[43] Matsuzawa Y, Funahashi T, Kihara S (2004). Adiponectin and metabolic syndrome. Arterioscler Thromb Vasc Biol.

[44] Xu H, Zhao Q, Song N (2020). AdipoR1/AdipoR2 dual agonist recovers nonalcoholic steatohepatitis and related fibrosis via endoplasmic reticulum-mitochondria axis. Nat Commun.

[45] Wei Y, Wang D, Topczewski F (2006). Saturated fatty acids induce endoplasmic reticulum stress and apoptosis independently of ceramide in liver cells. Am J Physiol Endocrinol Metab.

[46] Hu T, Wang J, Li W (2022). Endoplasmic reticulum stress in hepatitis B virus and hepatitis C virus infection. Viruses.

[47] Liu Q, Mu M, Chen H (2022). Hepatocyte steatosis inhibits hepatitis B virus secretion via induction of endoplasmic reticulum stress. Mol Cell Biochem.

[48] Chen H, Mu M, Liu Q (2020). Hepatocyte endoplasmic reticulum stress inhibits hepatitis B virus secretion and delays intracellular hepatitis B virus clearance after entecavir treatment. Front Med.

[49] Li J, Liu Y, Wang Z (2011). Subversion of cellular autophagy machinery by hepatitis B virus for viral envelopment. J Virol.

[50] Lin Y, Zhao Z, Huang A (2020). Interplay between cellular autophagy and hepatitis B virus replication: a systematic review. Cells.

[51] Tsai TH, Chen E, Li L (2017). The constitutive lipid droplet protein PLIN2 regulates autophagy in liver. Autophagy.

[52] Wang C, Gao XY, Han M (2023). Perilipin2 inhibits the replication of hepatitis B virus deoxyribonucleic acid by regulating autophagy under high-fat conditions. World J Virol.

[53] Huang SC, Su TH. (2025). Divergent roles of MASLD components in chronic hepatitis B: a double-edged sword. J Hepatol.

[54] Zhang S, Mak LY, Yuen MF (2025). Mechanisms of hepatocellular carcinoma and cirrhosis development in concurrent steatotic liver disease and chronic hepatitis B. Clin Mol Hepatol.

[55] Huang SC, Kao JH. (2026). Metabolic health in antiviral era of chronic hepatitis B: editorial on “Impacts of metabolic syndrome diseases on long-term outcomes of chronic hepatitis B patients treated with nucleos(t)ide analogues”. Clin Mol Hepatol.

